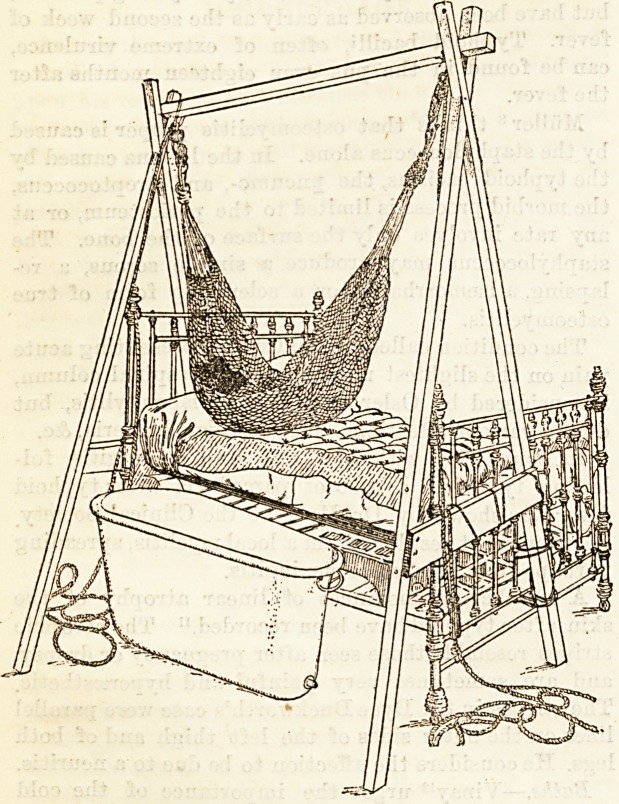# Typhoid

**Published:** 1894-03-31

**Authors:** 


### Medical Progress.

### TYPHOID.

*Hæmorrhage.*—Though the occurrence of intestinal hæmorrhage is always a cause of great anxiety in typhoid, many cases of it do as well as those in which there is none. Dr. Allan[1] points out that the diagnosis of hæmorrhage is by no means easy. There may be great restlessness, pallor, a fall of temperature, quick, soft pulse, and sometimes a curious earthy, raw-meat smell about the patient. Black motions may be ascribed to the effect of medicines, or a few drops of blood only may be passed, while the mass of what is lost may be retained in the bowel. Of course its possible source in epistaxis, hæmorrhoids, or menstruation must not be overlooked. He considers that the only remedies are stillness, iced drinks, and ice pads employed every alternate hour, turpentine, and lead and opium pills preferably cut into small pieces.

*General Treatment.*[2]—Even during an attack of typhoid, the need of preventing fresh infection from without through milk or water is considered of importance by Dr. J. M. G. Carter (Illinois). He lays stress also on the value of the cold bath with heart tonics, abundant nourishment from the beginning of the attack, active diuretics and diaphoretics, and mineral acids, especially the aromatic sulphuric, which aids both the gastric and pancreatic secretions. Similarly the administration of sulphurous acid[3] (♏ xx. tertiis horis) as an intestinal antiseptic is praised by Dr. W. Donovan.

In discussing the ordinary treatment Dr. G. Elliot[4] lays down that if symptoms show that a patient may be suffering from typhoid he should be treated at once as having it until he is well, or until typhoid can be excluded with certainty. He must be kept in bed on milk, and all reading, writing, and conversation prohibited. Four doses of calomel of from seven to ten grains, at forty-eight hours' interval, should be given in the first week, and one drop of carbolic acid with three of tincture of iodine in water every four hours throughout the disease. The treatment must be continued entire until the temperature has been normal day and night for a week.

It is worth noting[5] that in cases where the pyrexia is long-continued the evening temperature ceases to be higher than the morning. Where there are no other grave symptoms, this may be owing to a spread of the disease to fresh patches, but where pain and other abdominal symptoms also occur it is probably due to the enlargement of existing ulcers. Peritonitis is not always due to perforation, but occasionally to the rupture of a softened mesenteric gland, and the results of these cases are not so necessarily fatal. Among occasional complications it is well to remember the occurrence of convulsions, mania, ulceration of the larynx, and, especially if patients get about too soon, thrombosis of the femoral vein.

*Osteomyelitis.*[6]—A good deal of discussion has taken place with regard to osteomyelitis as a sequela. Chantemesse and Widal consider that the bacillus may cause apyretic chronic local affections of bone. The tibia is most frequently affected, either in the deeper layers of the periosteum or in the superficial layers of the diaphysis. The exostosis may sometimes be accompanied by pus.[7] The prognosis is invariably good, but there is always much pain. Usually a simple incision is all that is needed, but at times dead bone may require removal. The lesions may be multiple, and may not be detected for many months after the attack of typhoid, nor are they confined only to young patients, but have been observed as early as the second week of fever. Typhoid bacilli, often of extreme virulence, can be found in the pus even eighteen months after the fever.

Müller[8] thinks that osteomyelitis proper is caused by the staphylococcus alone. In the lesions caused by the typhoid bacillus, the pneumo-, and streptococcus, the morbid process is limited to the periosteum, or at any rate involves only the surface of the bone. The staphylococcus may produce a simple serous, a relapsing, a hæmorrhagic, or a sclerosing form of true osteomyelitis.

The condition called typhoid spine,[9] presenting acute pain on the slightest movement of the spinal column, is considered by Osler to be no perispondylitis, but due to various affections, such as a jar, hysteria, &c.

In connection with abscesses and meningitis following typhoid,[10] a case of paraplegia after typhoid fever was shown by Dr. Mott at the Clinical Society. He thought it resulted from a local myelitis, spreading perhaps from a patch of meningitis.

A number of instances of linear atrophy of the skin after typhoid have been recorded.[11] The atrophic stripes resemble those seen after pregnancy or dropsy, and are sometimes very painful and hyperæsthetic. The stripes in Sir Dyce Duckworth's case were parallel lines on the outer sides of the left thigh and of both legs. He considers the affection to be due to a neuritis.

*Baths.*—Vinay[12] urges the importance of the cold bath in the treatment of typhoid in pregnancy as lessening the maternal mortality from 17 to 6 per cent., without, however, greatly reducing the tendency to abortion. Vinary and Brand together had three deaths out of fifty-two cases. In mild typhoid abortion does not greatly increase the danger of the patient. It is generally preceded by a rigor and flooding, rise of temperature, with a subsequent fall. The cold baths must be continued after abortion if much pyrexia remains. Dr. G. C. Stephen[13] describes as follows an apparatus for moving typhoid patients in and out of cold baths. "I had fitted to the bed the apparatus shown in the illustration. The legs of the shears were tied to the foot of the bed, where they remained firmly in position. I attached—to use a sailor's expression—a "luff tackle purchase" to either end of the cross beam, and from the lower block of each purchase hooked the end of a common Indian hammock. By placing a sheet over the hammock, and spreading both out flat over the bed, the patient was able to roll on to it without inconvenience. Now by the multiplication of power effected by the combination of pulleys, the patient was raised off the bed with the greatest ease to the position shown in the illustration, and when suspended was readily pulled over the edge of the bed and gently lowered into the bath. In raising the patient out of the bath the pulleys were of great use. The hammock containing the sheet and patient was raised clear of the surface of the water, and retained in this position for a few seconds to allow it and the sheet to drip into the bath until fairly dry; after which it was raised to the level of the bed, and gently pushed over the edge on to a rubber sheet. The patient, being rubbed dry, is rolled into his original place at the further side of the bed, and the macintosh and wet sheet are removed."

*Antiseptics.*—Among those who have attempted the specific treatment of typhoid by bacterial products may be mentioned Fränkel,[14] who gave 57 patients sterilised injections of attenuated cultures grown on thymus bouillon. Considerable improvement was shown, " typhoid diarrhœa disappears, but relapses, and complications are not prevented." The fever was cut short and became remittent, copious perspiration and diuresis followed. The injections into the glutei muscles were continued at two days' intervals. The initial dose was ·5 c.cm., and convalescence was found to be very rapid. Rumpf used for fifty cases the products of B. pyocyanæus with similar results. Hammerschlag[15] used the blood of patients who had recently recovered, but with no very definite results.

Dr. Beavan Rake[16] has called attention to the difficulties of diagnosing typhoid in the West Indies, especially among dark skinned races, and where malarial fevers are common. He insists on the need of looking for the plasmodium in malaria, and thinks that typhoid may be spread by dust as well as by water. However, it is less common there than in temperate climates. The diagnosis from malarial fevers has caused much discussion in the south of the United States. Dr. Martin[17] considers that there is a third type lasting three to nine weeks, with nausea, costive bowels, and but little exhaustion. Microscopic evidence is wanting, but in certain of the doubtful fevers ulceration of Peyers' patches cannot be detected. The difficulty of distinguishing the typhoid bacillus from the B. coli communis has been discussed by McWeeney,[18] of Dublin, and Orlovsky[19] has shown that the former develops sulphuretted hydrogen chiefly when supplied with lead and iron salts, while the B. coli does so best with nitro-prussiate of sodium. Still this can hardly be relied on as a complete distinction at various stages of development. Another test for the two bacilli is given by Schild.[20] Tubes containing 1 in 7,000 of formalin are inoculated with pure cultures. If the B. coli is present they become turbid in the incubator in 24 hours, and the typhoid ones remain clear.

Ehrlich's[21] diazo reaction has been used in three thousand examinations by Friedenwald. He considers that Von Jaksch and others have failed from using too strong reagents, and the alcohol test, and from not saturating with ammonia. The red colour must be shown in the foam. He concludes that (1) The reaction is present almost without exception in typhoid, appearing in the first week and disappearing in the third, and does not justify a bad prognosis; (2) It does not occur in gastro-intestinal catarrh, healthy persons, or non-febrile diseases, malaria, or rheumatism, and rarely in other fevers; (3) in fatal cases of phthisis it is found for a long period.

1 Glasgow Med. J., Jan., 1894. 2 Int. Med. Mag., Nov., 1893. 3 B. M. J., Feb. 3, 1894. 4 Med. Rec., Nov. 18, 1893. 5 Clin. J., Dec. 17, 1893. 6 Med. Week, Dec. 6, 1893. 7 B. M. J., Jan. 6, 1894. 8 Münch. Med. Woch, 47 and 48, 1893. 9 Ann. J. Med. Sc.. Jan., 1894. 10 Lancet, Feb. 3, 1894. 11 J. Dermat., Dec., 1893. 12 Lyons Med., Dec. 3, 1893. 13 B. M. J., Jan. 20, 1894. 14 Med. Chronic, Dec.. 1893. 15 Med. Rec., Dec. 2, 1893. 16 B. M. J., Feb. 10, 1894. 17 Med. Rec., Dec. 16, 1893. 18 Lancet, Feb. 17, 1894. 19 B. M. J.. Feb. 17, 1894. 20 Centrall. f. Bakt., XIV., B. 22; and B. M. J., Feb. 3. 21 N. Y. Med. J., Dec. 23, 1893.

## Figures and Tables

**Figure f1:**